# Development and Validation of a Digital Image Processing-Based Pill Detection Tool for an Oral Medication Self-Monitoring System

**DOI:** 10.3390/s22082958

**Published:** 2022-04-12

**Authors:** Jannis Holtkötter, Rita Amaral, Rute Almeida, Cristina Jácome, Ricardo Cardoso, Ana Pereira, Mariana Pereira, Ki H. Chon, João Almeida Fonseca

**Affiliations:** 1 MEDCIDS—Department of Community Medicine, Health Informatics and Decision, Faculty of Medicine, University of Porto, 4200-450 Porto, Portugal; jannisholtkoetter@gmx.de (J.H.); rita.s.amaral@gmail.com (R.A.); ruteborgesalmeida@gmail.com (R.A.); cristinajacome.ft@gmail.com (C.J.); ambrpereira@gmail.com (A.P.); marianajoaopereira9@hotmail.com (M.P.); 2 CINTESIS—Center for Health Technology and Services Research, Faculty of Medicine, University of Porto, 4200-450 Porto, Portugal; 3 Department of Cardiovascular and Respiratory Sciences, Porto Health School, 4200-072 Porto, Portugal; 4 Department of Women’s and Children’s Health, Paediatric Research, Uppsala University, SE-751 85 Uppsala, Sweden; 5 MEDIDA—Serviços em Medicina, Educação, Investigação, Desenvolvimento e Avaliação, LDA, 4200-386 Porto, Portugal; ricnmc.94@gmail.com; 6 Department of Biomedical Engineering, University of Connecticut, Storrs, CT 06269, USA; ki.chon@uconn.edu

**Keywords:** computer vision, image processing, medication adherence, object detection, pill detection

## Abstract

Long-term adherence to medication is of critical importance for the successful management of chronic diseases. Objective tools to track oral medication adherence are either lacking, expensive, difficult to access, or require additional equipment. To improve medication adherence, cheap and easily accessible objective tools able to track compliance levels are necessary. A tool to monitor pill intake that can be implemented in mobile health solutions without the need for additional devices was developed. We propose a pill intake detection tool that uses digital image processing to analyze images of a blister to detect the presence of pills. The tool uses the Circular Hough Transform as a feature extraction technique and is therefore primarily useful for the detection of pills with a round shape. This pill detection tool is composed of two steps. First, the registration of a full blister and storing of reference values in a local database. Second, the detection and classification of taken and remaining pills in similar blisters, to determine the actual number of untaken pills. In the registration of round pills in full blisters, 100% of pills in gray blisters or blisters with a transparent cover were successfully detected. In the counting of untaken pills in partially opened blisters, 95.2% of remaining and 95.1% of taken pills were detected in gray blisters, while 88.2% of remaining and 80.8% of taken pills were detected in blisters with a transparent cover. The proposed tool provides promising results for the detection of round pills. However, the classification of taken and remaining pills needs to be further improved, in particular for the detection of pills with non-oval shapes.

## 1. Introduction

Worldwide, in 2019, patients collectively took medications on 1.8 trillion days, with a 3% annual increase since 2014. Pills are one of the most commonly prescribed formulations and one of the most preferred approaches by patients [1]. The preferred way of distributing prescribed medication in Europe (80%) is in the form of blister packs. Calendarized blister packs may improve compliance to prescribed medication by counteracting nonadherence factors, such as unintentional forgetfulness [2,3]. Moreover, blister packs allow third parties, for example, caregivers, to monitor the removal of the medication from the blister pack.

A crucial aspect of successful treatment is the patient’s adherence to prescribed medications. Poor adherence can diminish the effectiveness of the treatment plan and lead to severe risks for the patient. Yet, it is known that the adherence rate in patients with chronic diseases is low and approximately 50% of patients in developed countries do not take their medications as prescribed [4].

Assessment of adherence is an important aspect of a precise evaluation and adjustment of medical treatments. However, the measurement of adherence in the community is a difficult challenge since a “gold standard” method does not exist. Commonly employed subjective methods for adherence monitoring are either physician- or self-assessment, and pill counting. These methods are easy to implement and use, not very cost-intensive, and can achieve good results. However, they have also been shown to be imprecise and heavily dependent on the collaboration between patient and healthcare provider [5,6].

Electronic monitoring devices (EMDs) are a more objective method of adherence monitoring. Despite being proven to be highly accurate, the use of EMDs is limited in large populations due to high costs and possible practical issues, such as difficulties during the medication refilling in a local pharmacy [7]. Another straightforward and low-cost method that can be applied to different types of medication, such as tablets or inhalers, is dose counting, in which the number of doses taken is compared to the number of doses the patient has received, at the end of the analysis period. However, it only measures the average adherence and lacks information about daily adherence and other patterns of medication usage [8].

With the knowledge that nonadherence is a major concern in chronic care and that current methods for adherence monitoring are biased, imprecise, costly, and difficult to implement, new reliable methods, easy to implement and inexpensive, are needed. Smartphones accompany us in everyday life and therefore many applications, including for support of medication adherence, are being developed [7]. Examples of such applications are INSPIRERS-HTN, AIRDOC and InspirerMundi developed in collaboration with our team (https://paceit.med.up.pt/projects/, accessed on 25 March 2022). INSPIRERS-HTN is a mobile health application for the management of hypertension [9]. AIRDOC and InspirerMundi aim to support self-management for patients with chronic respiratory diseases [10,11]. These applications seek to provide cost-effective and easy-to-disseminate technological solutions, including treatment adherence assessment tools.

A typical modern smartphone is equipped with imaging sensors that can produce images of exceptional resolution and quality. Digital image processing has already played an important role in several projects related to the detection or identification of pills [12,13,14,15,16]. Lee and co-workers proposed an automatic pill identification and recognition program to identify illegal drugs [12]. In their proposal, an image of a pill is processed in several stages including preprocessing, edge detection, and feature vector construction. The extracted feature vectors are then compared to an existing pill database to create a ranking of pills that are most likely to match. Another mobile solution was proposed by Cunha et al. [13]. Their tool, HelpmePills, aims to support elderly people to prevent wrong ingestion by creating a personalized database of their medication history. The user is asked to place a pill on a detectable marker square and capture an image which is then automatically processed to extract information such as the shape, dimensions, and color of the pill. After creating a local database, the user can take a picture of the pill before the intake and the tool will compare it with the database for identification. The aforementioned pill identification tools require an initial database of pill features to correctly identify them. A project with the main focus on the development of an image processing software able to identify broken and unused pills in a blister pack for medication quality monitoring at production facilities was proposed by Rani et al. [14]. During the preprocessing stage, irrelevant details of the image, such as background and noise, are removed or at least reduced. Moreover, digital filters are applied to the image to highlight maximum intensity levels and detect edges that are created by the boundaries of the pills. The detected edges are converted into a binary image consisting of only the black and white pixels, in which ideally only the pill boundaries are displayed by white pixels. The binary image is then further analyzed for gaps between the detected edges using a horizontal and vertical histogram. These gaps should indicate the beginning and end of each individual pill boundary and therefore segment the image. Each individual pill segment is then used to extract features from it, which indicate if the pill is damaged, half-filled, or undamaged. The feature extraction is performed by correlation features, which describe the variation in the appearance of images of one type to another. Very encouraging experimental results of 96.33% accuracy were achieved over 50 blisters. In another research work, Sudharshan et al. present a system based on image processing techniques to detect damaged pills in tablet blisters during the pharmaceutical production process [15]. The system uses median filtering and bounding box techniques for the inspection of the blisters. Their system is designed for the inspection of blisters with red pills that are visible behind a plastic cover and can detect defective tablets with 95% accuracy. An approach for the detection and visualization of damaged tablets in the pharmaceutical industry was also proposed by Qasim and Al-Ani [16]. Their system includes image preprocessing techniques, feature extraction, and an artificial neural network algorithm (ANN) for the classification of tablets as damaged or undamaged. Total accuracy of 91.75% could be achieved for the detection of pills in a plastic cover blister. However, despite the proposal of several pill detection systems, no image-based solutions to measure medication adherence were found.

Since Duda et al. first outlined the use of the Hough transform to detect lines and curves in pictures [17] several Hough transform-based methods for circle detection have been proposed for a variety of image applications, including health-related ones [18]. Regarding the processing of medication blister images for quality control in the pharmaceutical industry, the usage of Circle Hough Transform (CHT) was proposed by Murai et al. [19] to detect both ends of the capsule region as circles, for visual inspection of medical capsules. In 2021, Zhao [20] used Canny edge detection and CHT to detect pills present in medication blister packs obtained by an industrial camera for quality control. Unfortunately, only illustrative results were published. More recently, Berciu et al. [21] proposed a method for identifying the correct cutting position of a pill, by means of the Hough transform, with 80% precision. The Circle Hough Transform (CHT) plays a major role in the methodology of our proposed round pill detection tool.

The objective of this research was to develop a pill detection tool able to count the number of remaining pills in a blister pack. The tool is intended to use images captured by smartphones in real-world conditions and to be compatible with as many pill blister types and shapes as possible, without the need to predefine a database for each blister brand. The ultimate goal is to provide a solution to the lack of validated pill detection tools able to objectively measure medication adherence measurement. We propose a new strategy for pills dispensed on blisters that constitutes an accessible, cheap, and easy-to-use pill detection tool, validated for round-shaped pills. To the best of our knowledge, our method is the first blister image-based solution, designed with the purpose to serve as objective adherence to medication measurement, with no need of a blister/pills database, and able to count present pills for any pill distribution on the blister. The tool developed in this project is designed keeping in mind the possibility of reuse in self-monitoring and management applications, such as the previously mentioned applications, INSPIRERS-HTN, AIRDOC and InspirerMundi.

The rest of this paper is constructed as follows: Section 2 describes the pipeline of the proposed pill detection tool, including the used image processing techniques. Section 3 presents the datasets that were used for development and testing of the proposed tool. Section 4 explains the validation strategy to test different functions of the tool. Section 5 presents the results of the tests and Section 6 discusses the results and presents possible future actions. The final Section 7 concludes this project.

## 2. System Overview

To cope with the goal to design a system compatible with variable pill blister types, we decided to consider a self-configuration approach. An initial image of the full blister will be used to extract a blister-specific configuration using the pill detection tool. Then, posterior images of the same or equal blisters are processed with respect to that configuration. Medication adherence can be estimated from successive detection of missing pills from the series of blister images.

**Figure 1:**
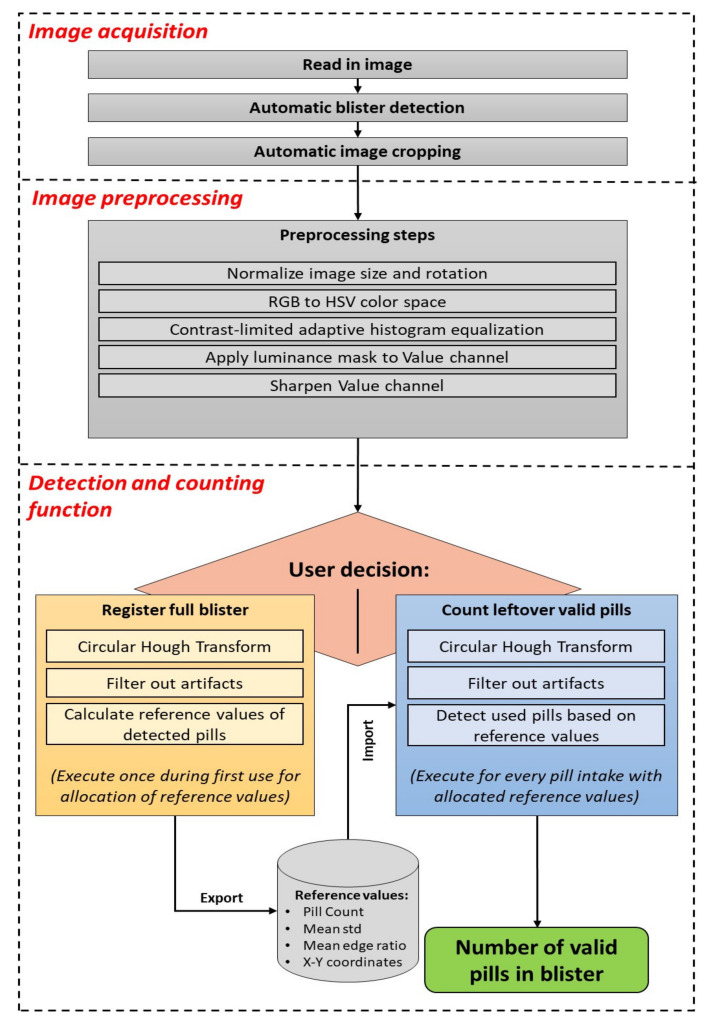
Pill detection tool pipeline.

The pill detection tool consists of three basic phases: image acquisition, image preprocessing, and detection and counting, as displayed in Figure 1. This prototype was developed with MATLAB software (The MathWorks Inc., Natick, MA, USA) on a computer for rapid development.

### 2.1. Image Acquisition

The first phase starts with the loading of an image of a pill blister into the pill detection tool. In the current offline MATLAB version, an image acquired using the smartphone camera is loaded for processing. Once implemented in a smartphone application, the user would have the possibility to either take an image with the smartphone camera or use a previously captured image from the image library. After the image is read in, it needs to be cropped in order to remove as much background from the image as possible and retain only the region of the blister. Ideally, the blister image is captured on a plain and flat background for a quick automatic blister detection to succeed.

**Figure 2:**
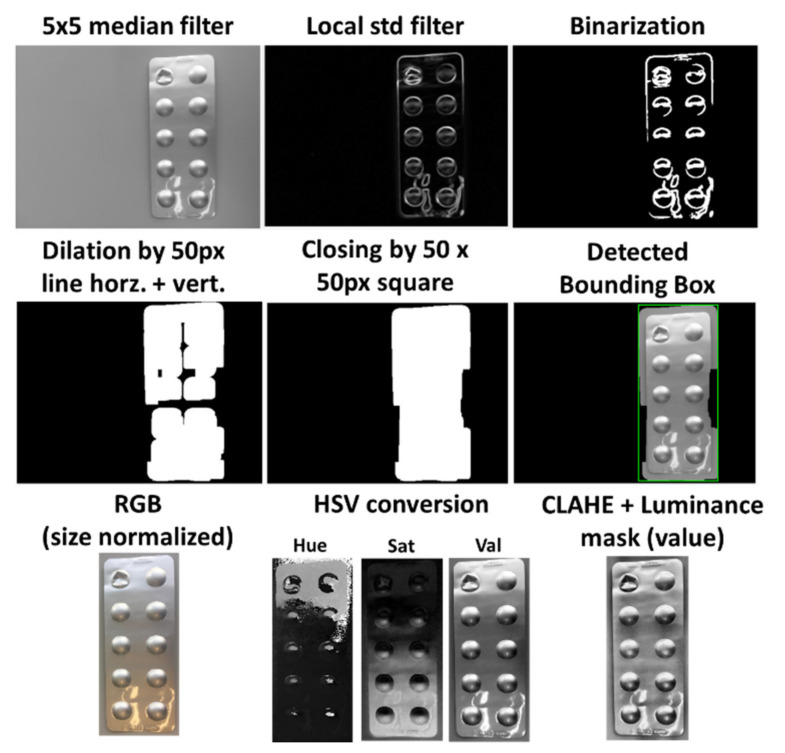
Individual steps of automatic blister detection and image preprocessing.

First, the image is converted into grayscale and filtered by a median filter with a neighborhood size of 5 by 5 pixels, which reduces noise and smooths the image. Then, the image is further filtered by a local standard deviation filter with a neighborhood size of 9 by 9 pixels to highlight contours and edges in the image. By using a global thresholding technique, the image is then transformed into a binary image, which displays heterogeneous areas as white objects, and homogeneous areas as the black background, as illustrated in the top row of Figure 2. The used global thresholding technique provided by MATLAB is based on Otsu’s method, which automatically calculates the threshold value for each image to minimize the intraclass variance between black and white pixels based on a 256-bin image histogram [22].

Most binary images at this step will contain several individual connected components, which often originate from the various contours on the blister. Furthermore, it is assumed that although the contours of the blisters are represented as individual connected components, they do not necessarily represent one single connected blister object. Moreover, there can also be objects in the background and general artifacts that disturb a homogeneous background of the binary image. To connect individual objects that have proximity to each other and to filter out possible confounding factors, morphological operations are employed as two consecutive dilations with a horizontal and vertical line of 50 pixels as a structural element, followed by a closing operation with a 50 by 50 pixels square as a structural element. Finally, the largest connected component in the image is chosen as the blister, since smaller objects or artifacts in the background could still be present after the initial morphological operations. The smallest enclosing rectangle, called the bounding box, is then proposed to the user as the blister. The progress of the morphological operations and the detected bounding box is displayed in the second row of Figure 2.

### 2.2. Image Preprocessing

The cropped image is then preprocessed to ensure that it has the required quality and comparability between multiple images of the same blister. First, the image size is scaled to 1000 pixels at the long edge while keeping the relation to the short edge. Additionally, the image is rotated as needed so that the blister is always vertically aligned. After this normalization, the image is converted from RGB (red, green, blue) into the HSV (hue, saturation, value) color space. Then, each channel is enhanced by contrast limited adaptive histogram equalization (CLAHE) to improve the local contrast of the images and enhance details’ definition, such as edges. The value channel plays an important role in the detection of edges in further processing functions. Thus, a luminance mask is applied to this channel to remove possible reflections from the image capturing process, which could distort the pixel intensities in the processing. Therefore, a region filling function based on inward interpolation is performed on all pixels over an intensity value of 0.95. Finally, the value channel is sharpened by a radius of 6 pixels and a strength of 60 to increase the detectability of edges and details in the image. The image preprocessing steps are illustrated in the bottom row of Figure 2.

### 2.3. Detection and Counting

The main step, which is responsible for counting the number of observed pills in the blister, consists of two separated branches: the registration and the counting modes. The basic concept of both branches is similar, however certain steps are executed differently. The registration mode is designed to detect all pills in full blisters from which no pill was dispensed yet, with no previous information available, and to retrieve reference values for that blister at the best possible conditions. The counting mode intends to detect and count remaining valid pills in a potentially (partially) used blister from the previously obtained reference values for that blister form at registration mode. Once the reference values for a type of blister are allocated, the counting mode can also be used to detect the number of pills in a full blister without allocating new reference values.

The registration mode begins with the detection of edges in the preprocessed value channel of the image, using the Canny edge detector [23]. This produces a binary image in which all edge classified pixels are displayed as white pixels and the rest as black pixels. After this edge detection, it is possible to detect circular shapes in the image with the use of the Circle Hough Transform (CHT). The CHT is a basic feature extraction function that uses a voting system in the Hough parameter space and the selection of local maxima in an accumulator matrix to detect circles [24]. For each detected circle, the radius, the center coordinates, and the metric value are noted. To mainly include actual pills only circles with a radius between 30 and 130 pixels are kept. The metric value ranges between 0 and 1, on which 1 expresses a high certainty of the detected object being circular. The sensitivity of the CHT was set by trial and error to 0.93, balancing between the inclusion of all pills and artifacts reduction.

It should be noted that round blister corners, structures on the blister, and reflections can introduce artifacts. Assuming that most detected circles are actual pills, as a full blister should be considered for registration mode, a combination of mean-based rules is used to discard false detections. The employed metric properties to reduce false detection consisted of the circle radius, metric value, deviation from median x-axis position, and the ratio of white pixels to a total number of pixels per circle, which we will refer to as “circle edge ratio”. These properties are analyzed and compared to their mean values for each detected circle in several conditional statements to determine outliers.

First, circles with a radius deviating more than 50% from the mean radius are classified as artifacts. Following, the circle edge ratio is used to further classify the remaining circles. To react dynamically to the appearance of the Canny edge detected image, the standard deviation of the circle edge ratio across all circles is calculated. A threshold is used to differentiate between two cases: high or low standard deviation of circle edge ratio. The threshold value was set to 0.04 and was estimated by trial and error. For the high case, the standard deviation is included in the upper and lower limit of the acceptable circle edge ratio. In the case of low standard deviation, the value itself is not used in the classification process because it tends to remove pills in images that have no more artifacts at this point. In both cases, high or low standard deviation, the metric value is considered if the mean is over 0.15, which indicates a certain informative value. A low metric mean value of under 0.15 shows that the metric value is not a good indicator to differentiate actual pills from artifacts. In case of a sufficient mean metric value, all circles with a metric value above 75% of the metric median are classified as pills and not as artifacts, even if the circle edge ratio indicates otherwise. This action should prevent an incorrect rejection of pills due to reflections or slight deformations of the package. Finally, all remaining circles are checked for their x-axis position in relation to the median x-axis position for the left and right half of the blister pack. Due to vertical rotation during the preprocessing steps, the pills in the blister should be in vertical columns. For the columns at most right and left sides of the blister, every pill that deviates from the median x-axis position by more than 5% of the image width is further checked regarding deviation distance, circle edge ratio, and metric value. The deviation limits for the circle edge ratio and metric value are stricter compared to the previous classification step. All identified outliers are considered artifacts and discarded. All remaining circles are identified as pills.

**Figure 3:**
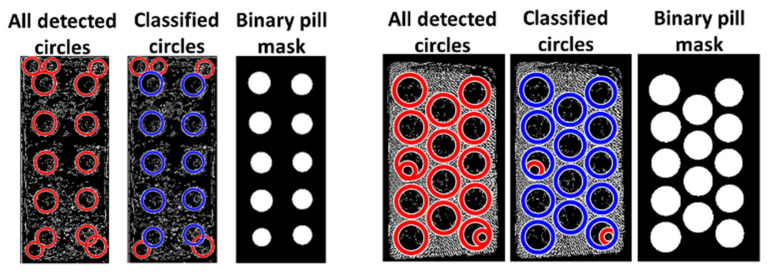
Two examples of the pill mask after classification of circles.

A binary mask of the reference identified pills is then created based on the center coordinates and radii of the classified pills, as illustrated in the two examples of a successful classification in Figure 3.

The final step of the registration mode is the calculation of reference pill values: mean radius, mean circle edge ratio, center-coordinates, and mean local standard deviation of all pills. The local standard deviation based on the pixel neighborhood represents the heterogeneity of pixel intensities in different regions. The mean of the local standard deviation for pixels within detected circles serves as a reference for detecting a pill.

The counting mode aims to detect and count the number of pills in an image of a blister that can be already partially used. Again, all circular shapes in the image are detected by a CHT and need to be then further classified into either an artifact or a detected pill. However, since empty pill pockets from taken pills are often detected as circular shapes, the mean property values, as used in the registration mode are not sufficient as comparison values for a pill that is present. Therefore, the detected circles are compared to the previously obtained reference values of the corresponding full blister.

First, for each position of the reference pills, the closest circle detected in the current image is determined by comparing the center coordinates. Considering a proximity as the mean pill radius plus 10 pixels, if more than one circle candidate is detected, the circles with the lowest deviation from the reference mean radius is chosen. This approach allows corresponding detected circles from registration to the ones in the opened blister, which originated from either a valid pill or from the empty pill pocket.

The comparison of coordinates assumes a certain level of similarity between current image and reference image regarding image crop. Blisters with an odd number of pills often have an asymmetrical pill alignment. The method of comparing the center coordinates from reference to the new image could produce misclassifications if the user rotated the blister by 180° concerning the image of the full blister. Therefore, if the full blister carries an odd number of pills, an additional step is added. For this, a copy of the preprocessed image is produced, rotated by 180° and the counting mode is applied. For each image version, original and rotated, the distance of each closest circle to the reference pills is added up and the version with the smaller total distance is selected for further use.

**Figure 4:**
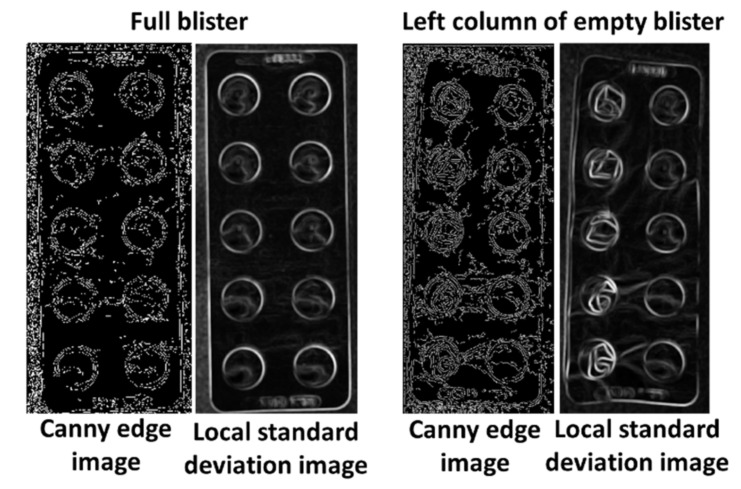
Intensity increase of local standard deviation and circle edge ratio due to deformation of pill pockets.

After the identification of the closest circles to the reference pills, the local standard deviation and the circle edge ratio of each circle are compared to the respective reference values. First, the local standard deviation is checked. An increase of 0.05 compared to the reference value leads to classification as a used pill. If the increase is lower than 0.05 but higher than 0.02 the circle edge ratio is consulted. If it shows an increase of at least 0.025 the pill in also classified as already used. These two properties are chosen because empty pockets should lead to an increase in the local standard deviation due to the higher heterogeneity of the surface and the deformations of the used pill pockets change the circle edge ratio. The appearance of these intensities in a blister with damaged and undamaged pill pockets is displayed in Figure 4. The remaining circles are labeled as present pills and used as the final count number.

Since the detection and counting mode is implemented in a two-step approach to provide self-configuration, the user must register a new blister at least once before the pill counting mode can be utilized. Important parameters are detected and stored in the registration step, which is required for the successful execution of the pill counting step.

## 3. Datasets

The dataset used during the development phase to determine metrics and thresholds consists of a total of 179 images of 9 different blisters. From those, 103 images display full blisters and were used to develop the registration mode. The other 76 images were used for the development of the counting mode and consist of 8 sequences, containing the full blister and a series of subsequent states in which a variable (not necessarily consecutive) number of pills is missing.

The testing dataset consists of a total of 280 images of 20 different blisters, taken in 31 sequences. Of the 20 different blisters, 9 blisters are the same as in the development dataset. Images with full blisters were used as a subset to test the registration mode (31 images). The sequences were used to test the counting mode (249 images).

The images in both datasets contain only blisters with round pill pockets in a variety of pill counts, and pills and blisters of different shapes and colors. The images were captured by several members of the research group with different smartphones in varying environments to simulate the variability of real-world blisters photographs taken by potential application users.

**Figure 5:**
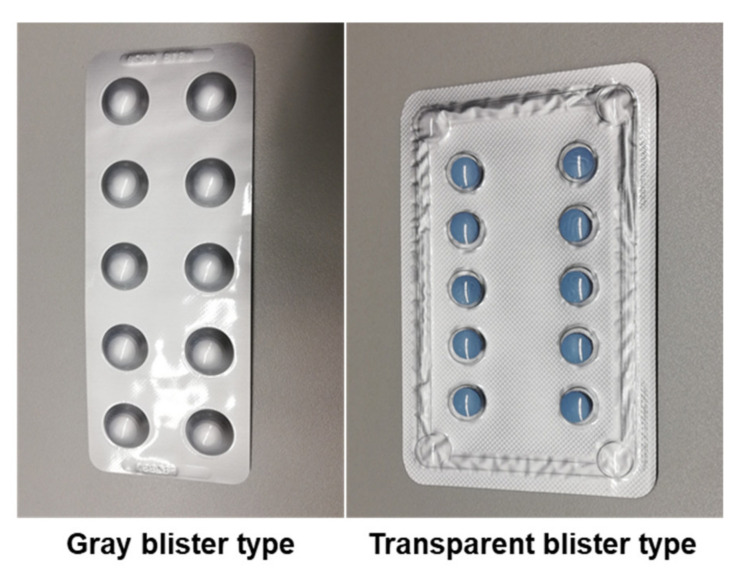
Examples of different blister types used in the datasets.

The blisters were categorized into two different types (Figure 5):

1. Gray: Blisters of gray color with gray pill cover;
2. Transparent: Blisters with a transparent pill cover, irrespective of the background color.

## 4. Validation Strategy

Three experimental validations were performed using varying subsets of the testing dataset. First, the automatic blister detection was tested with 60 images of 14 different, randomly chosen, blister brands. In 30 of these images, the blister was captured on a flat hand, and in the other 30 images, the blister was captured in front of a flat, plain background. Second, the registration mode was tested with the full blister reference image from each sequence, including 31 images. Third, the counting mode was tested with the full testing dataset of 249 images in 31 sequences. It should be noted that the 31 reference images that were used in the registration mode to prepare the reference values used in the counting mode, were not included in the validation of the counting mode. In the validation of registration and counting mode the images were cropped using the automatic blister detection. In all cases where the automatic blister detection produced insufficient results for further processing, the crop was adjusted manually.

The experimental validation plans are summarized in Table 1.

## 5. Results

First, the efficacy of the automatic blister detection was tested. Each blister detection was evaluated manually and specified as successful if the detected blister was positioned centered in the region, and the border around the blister did not exceed 1.5 cm. The automatic detection of blisters on a flat surface, like a table, was successful in 24 out of 30 images and failed 6 times due to edges in the background, or objects close to the blister. For blisters on the hand, the detection failed in 28 out of 30 images. For those images, the crop was adjusted manually.

The results of the registration mode test are displayed in Table 2. All pills in both blister types were detected. The number of false positives, in which an artifact is falsely identified as a pill, is slightly higher for transparent blisters: 0.4% compared to 0.2% in gray blisters.

The summary of the results for the counting mode is displayed in Table 3. The gray blister type showed an accuracy of 95.2%, which is over 10% higher than the accuracy for the transparent blister type.

**Figure 6:**
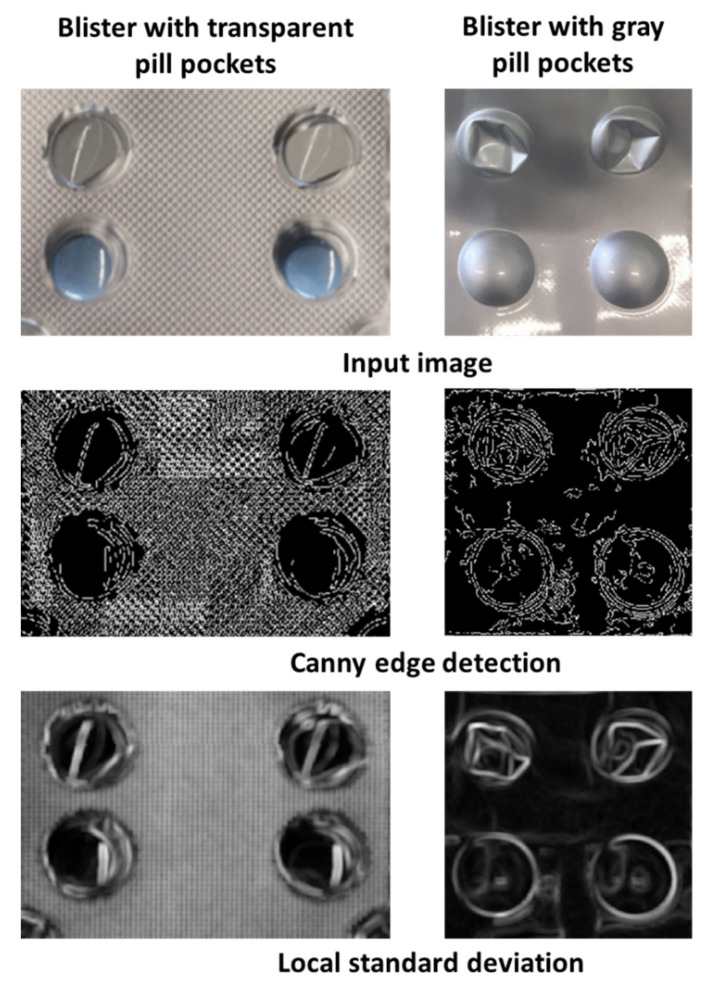
Comparison of transparent and gray pill pockets.

The difference between gray and transparent blister became very apparent in the image version filtered by the Canny edge detector or the local standard deviation (Figure 6). Edges and especially deformations in the area of the pill cover were much more visible in gray blisters due to the stronger contrast differences of the material.

## 6. Discussion

We proposed a digital image processing-based pill detection tool, which included function capabilities for automatic blister detection, registration of a full blister, and counting of present and missing pills in blisters that can be already partially used. The use of CHT in the registration and counting mode enabled accurate detection of pills in blisters with round pills. Keeping this in mind the dataset for the experimental validation was composed only of blisters with round pills. For the registration mode very good results, with almost perfect detection, were achieved. Good results were also achieved for the counting mode and our approach was better for blisters that cover the pill with a gray, opaque material than for those with a transparent pill cover. The results are promising and, although there is a need for additional improvement, this tool has the potential to be further developed and adapted for the modular implementation in future mobile health applications.

The automatic blister detection is intended to improve the experience since it requires less user input. The automatic blister detection showed acceptable results when the blister is placed on a flat surface like a table (80% successfully detected). The detection of the blister on the hand mainly fails due to the assortment of details and structures in the background. Therefore, the future recommendation for the user should be to place the blister on a table or other flat and homogeneous background. Since this is a simple instruction and the fulfillment of these requirements is relatively easy to comply with, this automatic blister detection tool is potentially useful to be used as the first step in pill detection. Nevertheless, it is also possible to include a functionality allowing the user to reject the automatic detection and use manual cropping instead. During the testing phase of registration and counting mode, we used the option of manual cropping, whenever the automatic blister detection produced insufficient results that could impact further processing. However, in the final version of the proposed pill detection tool, this optional feature would have to be analyzed regarding the usability for potential users of the application.

The experimental validation tests of the registration mode showed a correct pill detection rate of 100% for round pills in blisters of gray or transparent type. In addition, the low rate of false-positive detections (0.3% in total) indicates high accuracy for the detection of round pills, as expected due to the use of CHT. The small number of false positives can be allowed to be removed by the user in an additional verification step final to the registration of the blister. Moreover, the testing dataset consisted of only 31 images and needs to be further extended.

An important outcome of the registration mode is the precise location of the pills within the blister since this is an important asset for the extraction of pill features. There was the risk that too many artifacts would cause the mean property values to be skewed and therefore not give an estimation of the actual pill properties. Skewed mean property values would cause misclassification of the pills and artifacts. However, the results of the registration mode test for the gray and transparent blister show that a skewing of the mean property values was not an issue in the test dataset.

Subjective methods for adherence monitoring are either physician- or self-assessment, and pill counting. These methods are easy to implement and use, not very cost-intensive, and can achieve good results. However, they have also been shown to be imprecise and heavily dependent on the collaboration between patients and healthcare providers [5,6,7]. Usually, an adherence rate of 80% or more is required to consider good compliance with the therapeutic plan (medication adherence categories considered as low 0–50; medium 51–80 and high 81–100) [25]. Therefore, despite the accuracy not being at 100%, the results are still in an acceptable range for the measurement of adherence, as potential inaccuracies will still allow an adequate estimation. This represents an advantage with respect to the subjective methods currently used that have been shown to be highly unreliable, producing more than 30% of patients’ discordance in the medication adherence category between patients and physicians.

The counting mode of our proposed system used the values that were calculated for the individual pill regions with the registration step. It was able to detect 1158 out of 1267 circular pills (91.4%) present in the blisters. In blisters with gray, non-transparent pill cover accuracy of 95.2%, and in blisters, with transparent plastic pill cover, an accuracy of 84.5% was achieved. The total accuracy for all tested blisters was 89.6%. This result is not far from the results reported by Rani et al. [14] on 15 blisters with circular pills: 125 pills were detected out of 131 present (95.4%). In addition, they reported an accuracy of 98.4% for cylindrical and 93.75% for oval pills. However, the automated defective tablet detection tool from Rani et al. is adapted to the production process in pharmaceutical industries, where the image conditions are much more controlled and predictable compared to the image acquisition with a smartphone, regarding light/reflections, blister alignment in the image and presence of artifacts. Moreover, false-positive detections are not relevant for inspection and were not reported. Despite this, their proposed methodology is very interesting regarding a further improvement of our system, especially regarding the extension for the classification of non-circular pills. Sudharshan et al. [15] reported an accuracy of 95% with their proposed automatic detection tool of anomalies in blisters. In their methodology, the blisters are detected for colored regions using the different color channels of the image. This could be a beneficial approach for our system to improve the performance of colored pills behind a transparent plastic cover. However, there are no specifications regarding the size of the tested datasets or the variety of different blisters. Moreover, their tool is designed for the usage with blisters containing only red pills and is also intended for automatic quality control purposes during the manufacturing process. Qasim and Al-Ani [16] reported a total accuracy of 91.75% in 322 images with their proposed system which is using an artificial neural networking algorithm for the classification of a damaged and undamaged pill. Moreover, their system was designed for round or oblong-shaped pills in transparent plastic cover. However, as well as the previously mentioned systems, their proposed system is also developed for the implementation in the pharmaceutical production process, which means more consistent and controllable image quality, and a limited variety of blister types for which the method can be optimized. Nevertheless, their results are promising and show the potential for future inclusion of machine learning techniques in our proposed pill detection tool.

Due to the general lack of literature regarding pill detection in blister images acquired by smartphones in near real-world environments, an extensive comparison of the proposed results is limited.

The accuracy of the counting mode was higher for blisters that were covered by a gray, opaque material, than for blisters with a transparent pill cover. For the differentiation between damaged and valid pill pockets, values that are dependent on the heterogeneity and the visibility of edges were used. In blisters with gray pill pockets, deformations create clear edges with several strong contrast changes compared to undamaged pills. These edges become clearer after filtering by the Canny edge detector and local standard deviation. However, blisters with transparent pill pockets pose a difficult challenge for the detection of taken pills. Due to the transparent material deformations were more difficult to detect since edges and contrast changes were less visible. Therefore, the intensity increase for damaged pills is not that obvious in the filtered versions of the image and ultimately the damaged pill pockets remain undetected.

A possible solution to improve the detection of pill presence with a transparent cover could be the usage of different image channels, such as hue or saturation channels that create an image version based on the colors or saturation of the pixels. Since the pills are visible behind the transparent pill cover, the missing pill should show more clearly. However, using color has the disadvantage of also requiring you to extract the background color, as false detection can occur when it is visible through an empty pocket. The current version of the method does not depend on the color of the background.

Reflections, deformations of a valid pill slot, unsharp images, or objects in front of the blister, also caused misdetections. Reflections on the surface of the pills were influenced by several factors such as the lighting conditions of the image capture environment, the use of flash, and the material of the blister. Reflections on the pills can be interpreted as edges or deformations due to the induced contrast heterogeneity in the pill region. Therefore, valid pills with strong reflections can be misclassified as “taken”. The same issue arose for the detection of valid pills when deformations of the pill pocket were present. If the deformations caused an increase in the intensity of the local standard deviation and canny edge detected version of the image, it was likely that the same pill was misclassified as taken in every image of a blister sequence. Further, unsharp images usually produced multiple misdetections since the pill regions were not precise, and deformed pill pockets showed no clear difference from valid pill pockets because edges were blurred. Finally, another source of misdetection was the presence of interfering objects, such as a finger, that overlapped one or several pills in the images. The outline of the interfering objects in the region of the pill led to the misclassification of present pills as taken.

In general, the most important limitations and challenges of the proposed pill detection tool were the correct detections of valid and taken pills in blisters with transparent pill cover and the correction of several sources of misdetection. An approach for future improvement of the pill detection tool should include a further tuning of the reference values which are used to differentiate between taken and valid pills. The current reference values are static and only based on the mean of the reference image. Defining dynamic value ranges that rely on the overall change in intensity values within the image could improve the performance of the detection when changes in the image capture conditions occur. In addition, further transformations of the image should be tested to search for alternative metrics that are able to differentiate more clearly between valid and taken pills. Considering the specific purpose of adherence quantification, a further way to improve pill detection in a series of blisters could be the incorporation of data from previous detections of the same blister, complementing information obtained from the reference image. This could include the updated number and positions of pills from previous images or even the number of pills expected to be present according to the treatment plan.

Furthermore, different approaches to the problem of pill detection and counting could be considered. For example, machine learning methods can be explored in their ability to classify pocket pills as full or empty. Qasim and Al-Ani [16] have shown that machine learning can be an effective approach. However, it must be realized that the training of machine learning models requires large datasets that are composed of a wide variety of various types of blisters, requiring a much larger database. Different deep learning approaches for the identification of blister packages have been presented by Wang et al. [26] and Chung et al. [27]. Their datasets included over 17,000 and 30,000 images of over 200 different blister types. This further emphasizes the need to extend our dataset size to prepare for a future machine learning approach.

Finally, this pill detection tool needs to be extended to accommodate a detection mode for oblong pills. A base for this could be the automated tablet recognition tool, presented by Rani et al. [14], in which the vertical and horizontal boundaries in the image are identified to segment the individual pills and finally count the number of pills. Moreover, the defective pills are identified using correlation features that describe the variation among the appearances of the individual pills to each other, not assuming a particular shape contour, as does the CHT. The previously mentioned defective pill detection system by Qasim and Al-Ani [16] showed promising results for non-circular pills. Therefore, a machine learning approach might also provide a good solution for the extension of our proposed pill detection system to non-circular pills.

A summary of the limitations of our proposed pill detection tool and possible solutions which could be incorporated in a future work of our tool are presented in Table 4 below.

## 7. Conclusions

Our tool provides a well-performing pill detection for blister images captured by a smartphone in a near real-world environment. It requires the registration of a full blister and, afterward, uses extracted information for the counting of present pills in opened blisters. Despite the fact that only blisters with round pill shapes were covered by our tool, it provided a useful construct for further development and extension of functions, aiming at a future implementation in smartphone applications.

## Figures and Tables

**Table 1:** Experimental validation plans.

Test	Dataset	Test Description
Test 1 (Automatic blister detection)	*n* = 60 images/14 different blisters	Tests on images in different environments and counts how often the blister detection succeeded (subjective assessment).
Test 2 (Registration mode)	*n* = 31 images/20 different blisters/323 pills	Tests on images of full blisters and counts how many pills and false positives are detected.
Test 3 (Counting mode)	*n* = 249 images/20 different blisters/1267 pills	Tests on blisters with a varying number of present pills. Counts how many present pills are detected, and how many taken pills are correctly classified.

**Table 2:** Results of Test 2 (Registration mode).

	Blister Type		
	Gray	Transparent	Total
Number of images	17	14	31
Total tested pills	159	164	323
Total detected pills	162	170	332
False positives	3	6	9
Correct pill detections	159	164	323
Correct pill detection (%)	100%	100%	100%
False positives (%)	0.2%	0.4%	0.3%

**Table 3:** Results of Test 3 (Counting mode).

	Blister Type		
	Gray	Transparent	Total
Number of images	130	119	249
Total present pills tested	581	686	1267
Correct present pills detected	553	605	1158
Correct present pill detections (%)	95.2%	88.2%	91.4%
Total taken pills tested	659	682	1341
Correct taken pills detected	627	551	1178
Correct taken pills detections (%)	95.1%	80.8%	87.8%
Accuracy	95.2%	84.5%	89.6%

**Table 4:** Summary of limitations and possible solutions in a future approach.

Limitation	Possible Solution/Future Approach
Detection of missing pills behind transparent cover	Usage of different image channels, such as hue or saturation channel (see Sudharshan et al. [15])
Misdetections caused by reflections, dents in the pill pocket, unsharp images, or interfering objects in the image	Usage of more dynamic values rather than static values for classification of pills
Overall performance of counting mode	Usage of machine learning techniques for the classification process of present and absent pills (see Qasim and Al-Ani [16])
	Incorporation of information about the sequence (e.g., the number of pills that should be present in the blister according to the treatment plan or pill positions of the previous sequence image)
Lack of detection functionality for non-circular pills	Usage of vertical and horizontal boundary detection for pill segmentation in combination with correlation features (see Rani et al. [14])
	Usage of machine learning techniques for the classification process of present and absent pills (see Qasim and Al-Ani [16])

## Data Availability

The data presented in this study are available on request from the corresponding author.
